# The impact of prior SARS-CoV-2 infection on host inflammatory cytokine profiles in patients with TB or other respiratory diseases

**DOI:** 10.3389/fimmu.2023.1292486

**Published:** 2023-12-21

**Authors:** Annabelle Cottam, Ismaila L. Manneh, Awa Gindeh, Abdou K. Sillah, Ousainou Cham, Joseph Mendy, Amadou Barry, Edward G. Coker, Georgetta K. Daffeh, Simon Badjie, Salieu Barry, Olumuyiwa Owolabi, Jill Winter, Gerhard Walzl, Jayne S. Sutherland

**Affiliations:** ^1^ Faculty of Infectious and Tropical Diseases, London School of Hygiene and Tropical Medicine, London, United Kingdom; ^2^ Vaccines and Immunity Theme, Medical Research Council Unit The Gambia at the London School of Hygiene and Tropical Medicine, Fajara, Gambia; ^3^ Catalysis Foundation for Health, Emeryville, CA, United States; ^4^ Department of Molecular Biology and Human Genetics, University of Stellenbosch, Tygerberg, South Africa

**Keywords:** tuberculosis, COVID-19, inflammatory profiles, respiratory disease, cytokines

## Abstract

**Background:**

Tuberculosis (TB) and COVID-19 are the two leading causes of infectious disease mortality worldwide, and their overlap is likely frequent and inevitable. Previous research has shown increased mortality in TB/COVID-coinfected individuals, and emerging evidence suggests that COVID-19 may increase susceptibility to TB. However, the immunological mechanisms underlying these interactions remain unclear. In this study, we aimed to elucidate the impact of prior or concurrent COVID-19 infection on immune profiles of TB patients and those with other respiratory diseases (ORD).

**Methods:**

Serum and nasopharyngeal samples were collected from 161 Gambian adolescents and adults with either TB or an ORD. Concurrent COVID-19 infection was determined by PCR, while prior COVID-19 was defined by antibody seropositivity. Multiplex cytokine immunoassays were used to quantify 27 cytokines and chemokines in patient serum samples at baseline, and throughout treatment in TB patients.

**Results:**

Strikingly, TB and ORD patients with prior COVID-19 infection were found to have significantly reduced expression of several cytokines, including IL-1β, TNF-α and IL-7, compared to those without (p<0.035). Moreover, at month-six of anti-TB treatment, seropositive patients had lower serum Basic FGF (p=0.0115), IL-1β (p=0.0326) and IL-8 (p=0.0021) than seronegative. TB patients with acute COVID-19 coinfection had lower levels of IL-8, IL-13, TNF-α and IP-10 than TB-only patients, though these trends did not reach significance (p>0.035).

**Conclusions:**

Our findings demonstrate that COVID-19 infection alters the subsequent response to TB and ORDs, potentially contributing to pathogenesis. Further work is necessary to determine whether COVID-19 infection accelerates TB disease progression, though our results experimentally support this hypothesis.

## Introduction

Tuberculosis (TB), caused by *Mycobacterium tuberculosis* (*Mtb*), is one of the leading infectious disease killers worldwide, second only to coronavirus disease 2019 (COVID-19) (1). In 2021, there were 10.6 million new cases of TB and an estimated 1.6 million deaths, with low- and middle-income countries disproportionately affected (1). Due to disruptions in TB services caused by the COVID-19 pandemic, TB incidence and mortality are projected to further worsen in the next few years (2).

Anti-*Mtb* immunity relies on T-helper 1 (Th1) cells, which produce interferon (IFN)-γ, a type II IFN that activates macrophages and enhances anti-mycobacterial activity via a pro-inflammatory cytokine cascade (3). In 90% of cases, the *Mtb* infection is contained (termed latent TB) (4). However, when the host-pathogen balance changes, as with HIV co-infection, reactivation of the infection occurs, leading to active TB disease and associated inflammation (3). Although this inflammatory response is important for *Mtb* elimination, it contributes to tissue damage and lung pathology (5).

The causative agent of COVID-19 is severe acute respiratory syndrome coronavirus (SARS-CoV)-2 (6). As with *Mtb*, SARS-CoV-2 infection elicits a pro-inflammatory Th1-type cellular response (7). However, it also induces expression of type I IFNs, which play a central and varied role in anti-viral immunity (8). Notably, type I IFNs modulate the Th1/IFN-γ response to prevent hyperinflammation, though the extent to this negative feedback effect is context-dependent (9, 10). Clinically, cytokine storm and lymphopenia are hallmarks of COVID-19, correlating with disease severity (11).

As TB and COVID-19 both primarily affect the lungs, and have overlapping epidemiology and risk factors (12), research into how COVID-19 infection will affect TB pathogenesis is imperative. Despite poor characterization, a recent multi-country study found that TB is frequently diagnosed with or following COVID-19 (13). However, little is known about the impact of concurrent or prior COVID-19 infection on inflammatory profiles of TB patients, or those with other respiratory diseases.

A well-studied phenomenon is the suppression of protective *Mtb*-specific IFN-γ responses by virus-induced type I IFNs during co-infection. Despite their beneficial role in the anti-viral response, high levels of type I IFNs impair anti-*Mtb* Th1 cells and impede macrophage responsiveness to IFN-γ, resulting in decreased bactericidal activity and increased mortality (14, 15). Importantly though, the impact of viral infection on TB pathology extends beyond acute co-infection; prior influenza infection has been shown to cause immune dysregulation and subsequent enhanced susceptibility to TB, characterized by increased bacterial load and impaired treatment response (15).

Several studies have demonstrated that patients with TB and COVID-19 co-infection had higher morbidity and mortality than patients with TB or COVID-19 alone (16–18). It has since been found that co-infected patients have reduced *Mtb*-specific Th1 cells (19). Therefore, it is reasonable to assume that *Mtb* and SARS-CoV-2 infections synergistically lead to poorer outcomes, potentially through immune dysregulation as found with previous viral-TB co-infections. Interestingly, emerging clinical and epidemiological data suggest that COVID-19 infection increases susceptibility to TB (20, 21). Nonetheless, the immunological mechanisms behind these findings remain unclear, probably due to the novelty of SARS-CoV-2 and the indistinguishable symptoms of TB and COVID-19, leading to underreporting of cases. The aim of this study was to assess the impact of both concurrent (through PCR detection) and prior (through serology) SARS-CoV-2 infection on the serum inflammatory cytokine profiles of TB patients and patients with other respiratory diseases.

## Materials and methods

### Ethical approval

Ethical approval was obtained from the LSHTM Ethics Committee and the Gambia Government/MRCG Joint Ethics Committee (LEO: 21727). Written informed consent was obtained from all participants; children signed assent forms and were consented for by their parent or guardian.

### Participants

Adolescents and adults who presented at the MRC clinic in The Gambia with symptoms suggestive of TB, but prior to microbiological confirmation, were recruited into the study. Symptoms included a cough lasting for over two weeks, plus either fever, malaise, unexplained weight loss, night sweats, hemoptysis, chest pain, or decreased appetite. Exclusion criteria included unwillingness to be tested for HIV, pregnant or breastfeeding women, clinical pallor or other signs of severe anemia, current systemic steroid use or immunosuppressive therapy in the past four weeks, and those on TB treatment or isoniazid preventive therapy currently or in the last 90 days. Patients who were confirmed to have TB were referred to the National TB Program for treatment initiation and were followed to completion of treatment.

### Clinical characterization and sample collection

All patients provided sputum samples, which were analyzed using GeneXpert Ultra (Cepheid, USA). Patients who tested positive for *Mtb*, as well as those who tested negative but received a clinical TB diagnosis, were referred for anti-TB treatment. The remaining *Mtb*-negative participants were classified as having another respiratory disease (ORD). All patients also had a nasopharyngeal swab collected at time of enrolment to test for COVID-19 infection using PCR-based SARS-CoV-2 Xpert Xpress cartridges (Cepheid, USA). Additionally, serum samples were collected and tested for anti-SARS-CoV-2 spike protein antibodies by ELISA or rapid diagnostic test (NowCheck, South Korea). Those who tested PCR-positive were defined as having acute COVID-19 co-infection and those who were antibody-positive (seropositive) were classified as having had prior COVID-19 infection. Rapid HIV testing was also carried out on all participants.

Serum samples were collected from all participants at the initial screening stage and from TB-positive individuals at week 4, 8, 16 and 24 of anti-TB treatment. Samples were stored at -80°C until analysis.

### Quantification of serum cytokines using multiplex immunoassay

Bio-Plex Multiplex Pro Human Cytokine Assay kits (BioRad, Belgium) were used for the quantification of 27 different cytokines in serum samples, according to the manufacturer’s instructions. Analytes measured were Basic FGF, Eotaxin, G-CSF, GM-CSF, IFN-γ, IL-1β, IL-1ra, IL-2, IL-4, IL-5, IL-6, IL-7, IL-8, IL-9, IL-10, IL-12, IL-13, IL-15, IL-17, IP-10, MCP-1, MIP-1α, MIP-1β, PDGF-BB, RANTES, TNF-α and VEGF.

Briefly, frozen samples were thawed overnight and centrifuged at 600g for 15 minutes at 4°C to remove particulate matter. The assay beads were vortexed and diluted to 1x in assay buffer, and 50μl was added to each well. Next, 50μl of undiluted serum samples were added, along with 50μl of reconstituted standards, controls and blanks, and the plate was incubated on a shaker (at 850 ± 50 rpm) for 30 minutes. The wells were then incubated with 25μl of 1x diluted detection antibodies for 30 minutes and 50μl of 1x diluted streptavidin-phycoerythrin for 10 minutes. Washes were performed after each step. Finally, the beads were resuspended and shaken in 125μl of assay buffer. The plate was read using the Bio-Plex 200 System. Cytokine concentrations were calculated based on the standard curves generated for fluorescence intensity vs pg/mL. Standards and controls were run in duplicate.

### Statistical analysis

Cytokine concentration values below the limit of detection (out of range (OOR<)) were replaced with half the minimum expected standard concentration for the respective cytokine. Similarly, those OOR> were substituted with 2X the top standard. Statistical analysis was performed using GraphPad Prism version 8.4.1 (Software MacKiev, USA). Baseline study group comparisons were performed using two-tailed Mann-Whitney tests. For the longitudinal analysis, a Kruskal-Wallis test was used, with Dunn’s multiple comparisons test to analyze differences between timepoints. Statistical significance was defined as a p-value of ≤ 0.035 after adjusting for false discovery rate.

## Results

### Participant demographics and clinical features

161 participants were included in our study. Of these, 68 (42.2%) were diagnosed with TB, either microbiologically (n=62) or clinically (n=6), and 93 (57.8%) were diagnosed with an ORD. The ORD diagnoses mainly consisted of infection (44%), allergy (40%) or chronic lung disease (7%). Participants were further divided according to COVID-19 PCR and serology results. 49% of PCR-negative TB patients and 65% of PCR-negative ORD patients were seropositive for COVID-19; thus, to avoid any influence on results, these participants were excluded from the PCR-negative groups. Likewise, PCR+ participants were excluded from seropositive and seronegative cohorts for analyses (see **Supplementary Figure 1** for participant flowchart).

The median age [interquartile range (IQR)] of TB patients was 29.5 years [23-39], while in ORD patients, it was 32 [25-44] (p=0.0967). Notably, 83.8% of TB patients were male, whereas only 53.8% of ORD patients were male (p<0.0001). Seropositive TB patients were significantly younger than seronegative, with median [IQR] ages of 26 [21-34] and 36 [27-43] years, respectively (p=0.0025). However, there were no other significant differences in age, sex ratio or proportion of HIV+ individuals between comparison groups (**Table 1**).

**Table 1:** Participant Demographic Characteristics.

	TB				ORD			
	PCR +	PCR -	Sero +	Sero -	PCR +	PCR -	Sero +	Sero -
**n**	5	24	39	24	8	25	60	25
**Median age [IQR]**	23.0 [20-38]	36.0 [27-43]	26.0 [21-34]	36.0 [27-43]	37.5 [30-45]	32.0 [24-45]	31.5 [25-43]	32.0 [24-45]
**Males, n (%)**	5 (100)	21 (87.5)	31 (79.5)	21 (87.5)	6 (75.0)	13 (52.0)	32 (53.3)	13 (52.0)
**HIV, n (%)**	0	2 (8.3)	0	2 (8.3)	0	4 (16.0)	2 (3.3)	4 (16.0)
***Mtb-*confirmed, n (%)**	4 (80.0)	20 (83.3)	38 (97.4)	20 (83.3)	–	–	–	–

n, sample size; IQR, interquartile range; HIV, Human Immunodeficiency Virus; *Mtb, Mycobacterium tuberculosis*; TB, Tuberculosis; ORD, other respiratory disease; PCR, Polymerase Chain Reaction for SARS-CoV-2 antigens; Sero, serology test for SARS-CoV-2 antibodies.

### Baseline serum cytokine levels in TB and ORD patients

**Figure 1:**
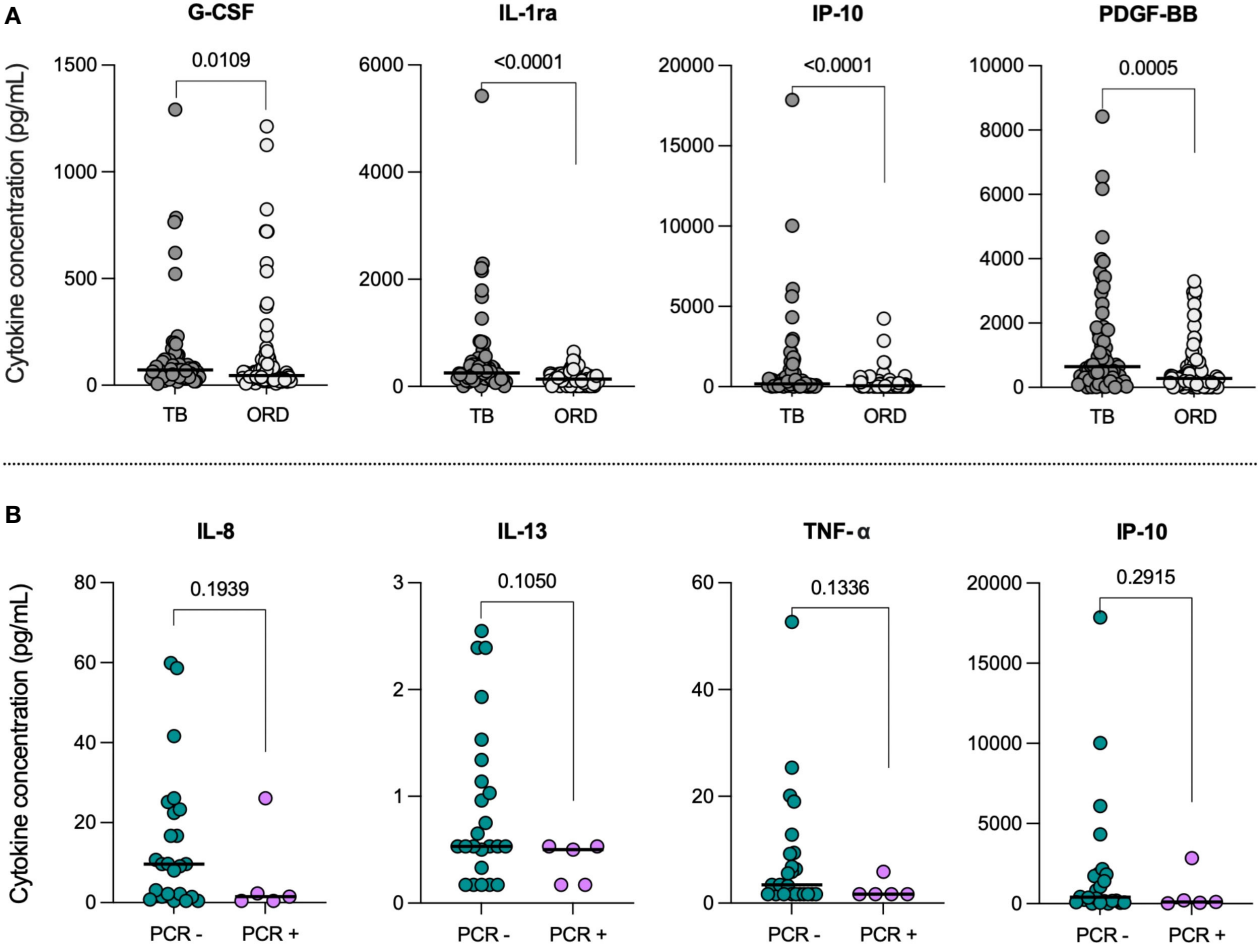
**(A)** Baseline serum cytokine concentrations in TB and ORD patients. Scatter plots illustrate the distribution of serum concentrations (pg/mL) of G-CSF, IL-1ra, IP-10 and PDGF-BB in the TB (n=68) and ORD (n=93) cohorts. Median concentrations are depicted as solid lines. Comparisons were performed using Mann-Whitney tests. **(B)** Serum cytokine concentrations in TB patients with (PCR+) and without (PCR-) concurrent COVID-19. Scatter plots illustrate the distribution of serum IL-8, IL-13, TNF-α and IP-10 concentrations (pg/mL) within PCR- (n=24; green) and PCR+ (n=5; pink) TB cohorts. Medians are represented by solid lines. Comparisons were performed using Mann-Whitney tests.

To compare baseline inflammation levels in TB and ORD patients, we first analyzed the difference in serum cytokine concentrations between the two groups, regardless of COVID-19 status (**Figure 1A**). Of the 27 cytokines analyzed, four were differentially expressed in TB and ORD participants. Levels of G-CSF (p=0.0109), IL-1ra (p<0.0001), IP-10 (p<0.0001) and PDGF-BB (p=0.0005) were all found to be significantly lower in ORD patients, compared to those with TB.

### Analysis of serum cytokine levels in TB and ORD patients with concurrent COVID-19 disease

To determine the impact of acute COVID-19 co-infection, we first compared serum cytokine levels in co-infected (PCR+; n=5) and mono-infected (PCR-; n=24) TB patients. IL-8, IL-13, TNF-α and IP-10 showed trends towards lower levels in COVID+ TB patients, compared to those with TB alone (**Figure 1B**); however, statistical significance was not reached. Notably, the decrease in median IL-13 from 0.53pg/mL [IQR: 0.37-1.29] in the TB-only group to 0.50pg/mL [0.17-0.53] in the TB/COVID-19 group had the lowest p-value (p=0.1050). No differences were observed between ORD patients with (PCR+; n=8) and without (PCR-; n=25) acute COVID-19 infection.

### COVID-19 seropositivity is associated with reduced serum cytokine levels in TB patients

**Figure 2:**
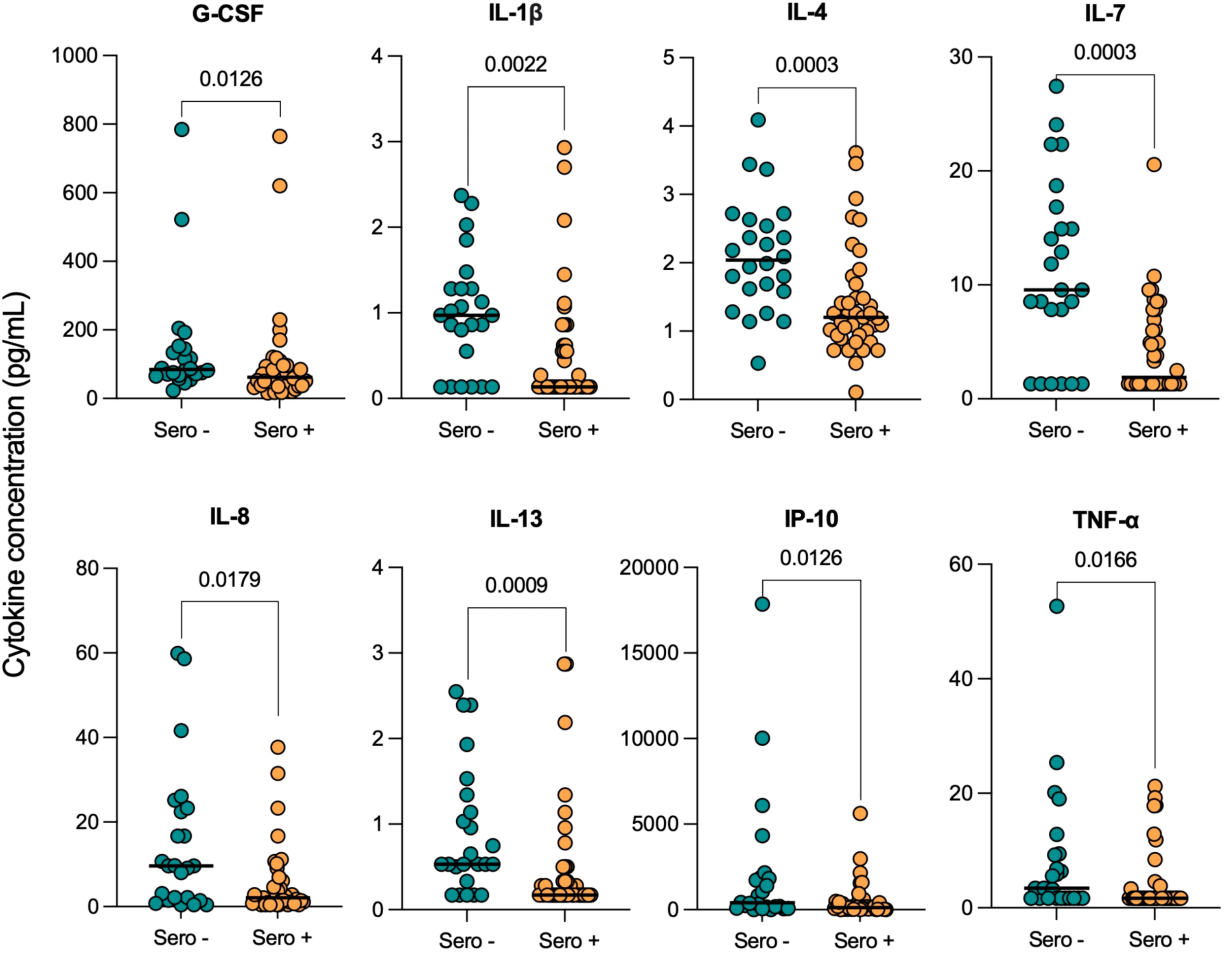
Serum cytokine concentrations in TB patients with (Sero+) and without (Sero-) prior SARS-CoV-2 infection. Scatter plots represent the distribution of serum cytokine concentrations (G-CSF, IL-1β, IL-4, IL-7, IL-8, IL-13, IP-10, TNF-α) (pg/mL) within SARS-CoV-2 Sero+ (n=39; orange) and Sero- (n=24; green) TB patient cohorts. Medians are displayed as solid lines. Comparisons were performed using Mann-Whitney tests.

Serum cytokine levels were next compared in TB patients who had prior SARS-CoV-2 infection (Sero+; n=39) to those who hadn’t (Sero-; n=24), at the time of TB diagnosis. Interestingly, we found consistently decreased cytokine and chemokine levels in TB patients with prior COVID-19 (**Figure 2**).

Serum concentrations of G-CSF (p=0.0126), IL-1β (p=0.0022), IL-4 (p=0.0003), IL-7 (p=0.0003), IL-8 (p=0.0179), IL-13 (p=0.0009), IP-10 (p=0.0126) and TNF-α (p=0.0166) were all significantly lower in SARS-CoV-2 seropositive TB patients compared to seronegative. Notably, the decreases in median IL-4 (2.04 [1.6-2.6] vs 1.20 [0.9-1.7]), IL-7 (9.55 [2.9-16.4] vs 1.89 [1.3-7.2]) and IL-13 (0.53 [0.4-1.3] vs 0.17 [0.2-0.5]) concentrations (pg/mL) were highly significant (p<0.001). Median serum concentrations of differentially expressed cytokines and their corresponding p-values are displayed in **Supplementary Table 1**. The remaining cytokines were either undetectable in the majority of participants, such as IFN-γ, or were not significantly impacted by COVID-19 serostatus, such as IL-1ra.

### Decreased serum cytokine concentrations in ORD patients with prior COVID-19

**Figure 3:**
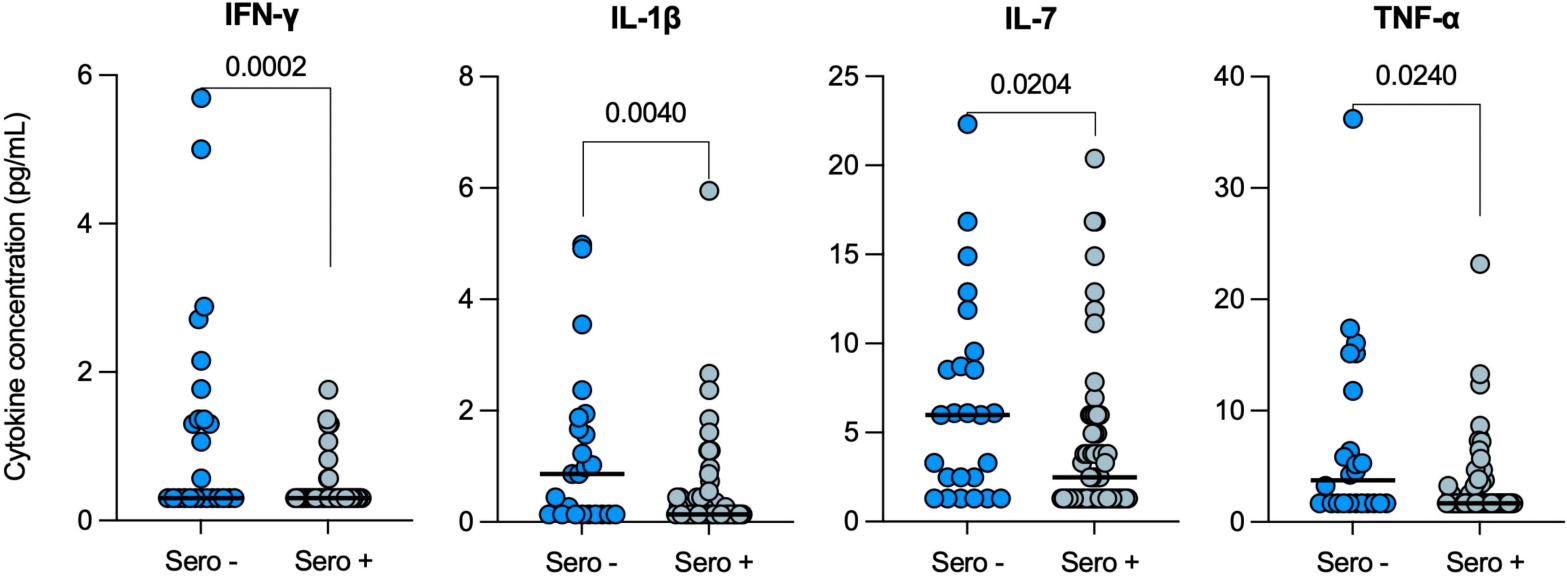
Serum cytokine levels in ORD patients with (Sero+) and without (Sero-) prior SARS-CoV-2 infection. Scatter plots represent the distribution of serum cytokine concentrations (IFN-γ, IL-1β, IL-7 and TNF-α) (pg/mL) within SARS-CoV-2 Sero+ (n=60; light blue) and Sero- (n=25; dark blue) ORD patient cohorts. Medians are represented by solid lines. Comparisons were performed using Mann-Whitney tests.

Serum cytokine levels were then compared in ORD patients with (Sero+; n=60) and without (Sero-; n=25) evidence of prior SARS-CoV-2 infection, and four cytokines were found to be significantly lower in seropositive ORD patients: IFN-γ (p=0.0002), IL-1β (p=0.0040), IL-7 (p=0.0204) and TNF-α (p=0.0240) (**Figure 3**). In particular, the decrease in median IFN-γ from 0.3pg/mL [0.3-1.6] in the seronegative group, to 0.3pg/mL [0.3-0.3] in patients with prior COVID-19 was highly significant (p<0.001).

### COVID-19 infection alters cytokine normalization during TB treatment

**Figure 4:**
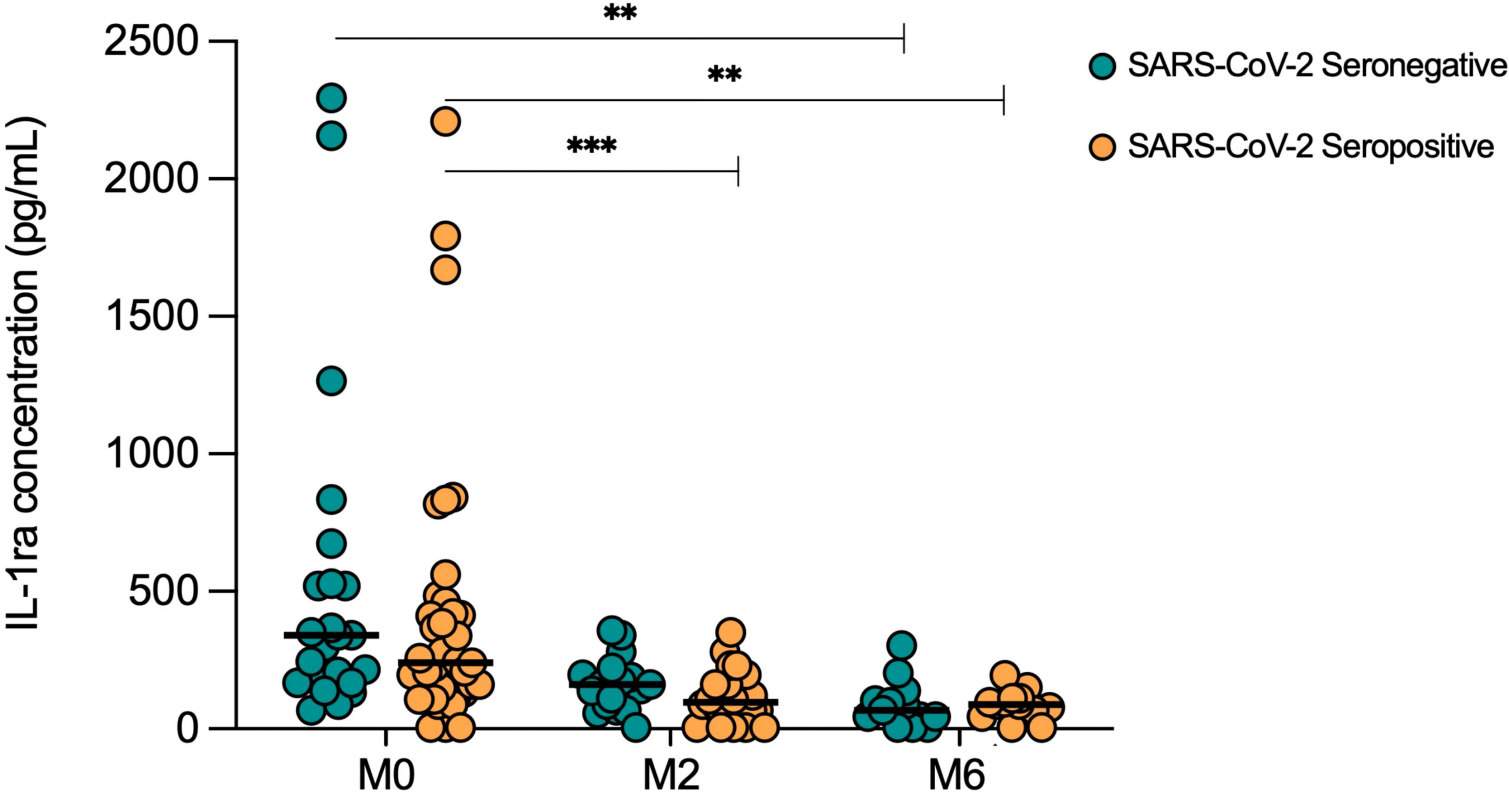
Serum IL-1ra levels in TB patients with (Sero+) and without (Sero-) prior SARS-CoV-2 infection throughout TB treatment. Longitudinal scatter plots illustrate the distribution of serum IL-1ra concentration (pg/mL) within SARS-CoV-2 Sero- (n=24; green) and Sero+ (n=39; orange) TB patient cohorts. Medians are represented by lines. (** p<0.001 *** p<0.0001). A Kruskal-Wallis test was used to analyze trends in cytokine distributions, with Dunn’s multiple comparisons to test for differences between timepoints.

To investigate the influence of COVID-19 on response to TB treatment, serum cytokine levels were longitudinally measured in TB patients with (Sero+; n=39) and without (Sero-; n=24) prior SARS-CoV-2 infection, throughout six months of anti-TB treatment. IL-1ra concentrations decreased from screening to month 6 of treatment in both SARS-CoV-2 seronegative (p=0.0004) and SARS-CoV-2 seropositive (p=0.0003) TB patients to a similar degree (**Figure 4**). Of note, in the seropositive TB group, IL-1ra levels were also significantly lower than screening at month 2 (p=0.0008) and month 4 (p<0.0001; data not shown) of treatment.

**Figure 5:**
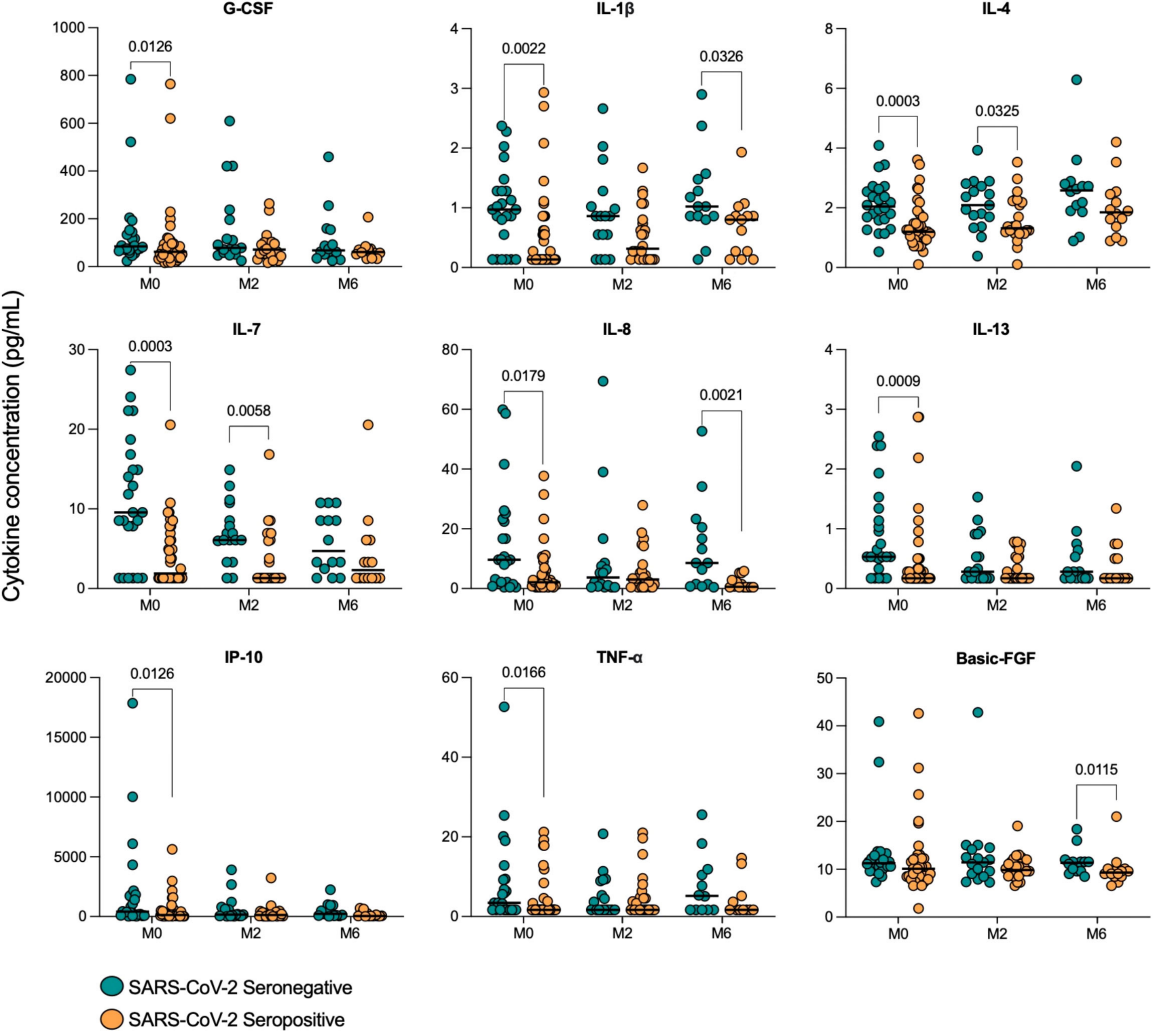
Serum cytokine concentrations in TB patients with (Sero+) and without (Sero-) prior SARS-CoV-2 infection throughout TB treatment. Scatter plots show the distribution of cytokine concentrations (G-CSF, IL-1β, IL-4, IL-7, IL-8, IL-13, IP-10, TNF-α and Basic FGF) (pg/mL) in SARS-CoV-2 Sero- (n=24; green) vs Sero+ (n=39; orange) TB patients at months (M) 0, 2 and 6 of TB treatment. Comparisons were performed using Mann-Whitney tests.

When SARS-CoV-2 seropositive and seronegative TB patients were compared at month 2 of treatment, both IL-4 (p=0.0325) and IL-7 (p=0.0058) remained lower in seropositive patients compared to seronegative. At month 6 (treatment endpoint), three cytokines were found to be significantly lower in SARS-CoV-2 seropositive TB patients than seronegative: Basic FGF (p=0.0115), IL-1β (p=0.0326) and IL-8 (0.0021) (**Figure 5**).

## Discussion

Despite the global importance of both TB and COVID-19, little is known about the immunopathology underlying their interaction. In this study, we investigated the impact of prior (seropositive) or concurrent (PCR+) COVID-19 infection on the serum inflammatory cytokine profiles of patients with TB and those with ORD. To our knowledge, this is the first study to characterize the impact of prior COVID-19 on host inflammatory markers in TB or ORD patients. Remarkably, we found consistently reduced cytokine expression in TB and ORD patients with prior SARS-CoV-2 infection, suggesting that COVID-19 has a long-term impact on the subsequent response to both TB and non-TB respiratory diseases.

TB patients with acute COVID-19 had lower levels of IL-8, IL-13, TNF-α and IP-10 compared to those with TB alone, although these trends did not reach significance. Research has shown upregulation of these cytokines in both TB and COVID-19 patients relative to healthy controls, suggesting an immunoregulatory effect of co-infection (22–24). These results support a previous finding that COVID-co-infected patients had reduced *Mtb*-specific T-cells, as IL-13, TNF-α and IP-10 are produced by Th1/Th2 cells in TB (19, 25). Consistently, a recent study found lower Th1- and Th2-associated cytokines, including IP-10, in co-infected patients compared to TB-only patients (26); however, a concomitant rise in innate inflammation was reported, which we did not see. Differences in baseline inflammation and pathogen genetics may explain this discrepancy, as their participants were mostly from a non-endemic setting. Moreover, SARS-CoV-2 seropositive participants were excluded from our TB-only group to provide a clean comparison against COVID+ TB patients. Nonetheless, our study was limited by a small sample size in the co-infected group, so larger numbers are needed to achieve significance and confirm our preliminary findings. Of note, IP-10 is directly induced by IFN-γ (22), hence its decrease in co-infection may indicate regulation by type I IFN, in line with other viral-microbial co-infections. Future studies should measure both type I and II IFNs to assess for a negative correlation.

Unexpectedly, we found that TB patients with prior COVID-19 infection had consistently decreased cytokine expression compared to those without. Only 13% of our participants were vaccinated against COVID-19, but 66% tested seropositive, hence vaccination status is unlikely to have influenced serology results. Many of the cytokines that were downregulated in SARS-CoV-2 seropositive TB patients are critical in anti-*Mtb* immunity, such as TNF-α, IP-10 and IL-1β, which coordinate immune cell activation and granuloma formation (25). COVID-19 infection may therefore impair the subsequent response to *Mtb* and increase bacterial burden, however, it might simultaneously reduce inflammation-associated lung pathology. Importantly, these findings may indicate an increased risk of TB disease progression following SARS-CoV-2 infection, supporting preliminary clinical and epidemiological concerns that COVID-19 increases susceptibility to TB (21), though, this can only be determined in a longitudinal cohort study.

Several studies have demonstrated immune cell dysregulation following acute COVID-19 (27, 28). Winheim et al. found that patients had long-term depletion of dendritic cells and monocytes, and those that did regenerate were less able to be stimulated (28). Furthermore, decreases in both naïve and helper T-cells can reportedly persist for several months post-acute COVID-19 (29, 30). These immunological sequelae provide a plausible explanation for the reduced cytokine expression observed in our SARS-CoV-2 seropositive TB patients: TNF-α, G-CSF, IL-1β and IL-8 are produced by myeloid cells in response to *Mtb* stimulation, and IP-10, IL-4 and IL-13 are released by T-cell subsets in TB (25). Interestingly, a recent study found that COVID-19 patients had elevated serum IFN-β (type I IFN) for up to eight months following infection, suggesting possible sustained modulation of the IFN-γ response (29). Flow cytometry would provide more insight into the characteristics of cytokine-producing cells in SARS-CoV-2 seropositive TB patients.

COVID-19 seropositivity was also associated with reduced pro-inflammatory cytokine expression in patients with non-TB respiratory diseases. Consistent with our findings in TB patients, prior COVID-19 led to reduced levels of IFN-γ, IL-1β, IL-7 and TNF-α in ORD patients. This may reflect that the same immune cells were impacted by prior SARS-CoV-2 infection, further supporting that COVID-19 causes a long-lasting distinct type of immune dysregulation. However, several markers that were downregulated in seropositive TB patients were not affected by seropositivity in ORD patients, including IP-10, IL-4 and IL-13. TB patients had higher baseline IP-10 than ORD patients, implying IP-10 is more relevant in the TB response and thus impacted to a greater degree. Though, baseline IL-4 and IL-13 levels were similar in TB and ORD cohorts. Both cytokines can be released by granulocytes in ORDs such as allergic asthma, which according to Winheim et al., were not affected by COVID-19 (28, 31). Nonetheless, our study was limited by a lack of characterization of ORDs. It would therefore be interesting for future studies to distinguish the impact of prior COVID-19 on infectious versus non-infectious respiratory diseases.

Having established that prior COVID-19 alters immune profiles in TB patients, we went on to investigate whether this influenced the immune response to TB treatment. Both SARS-CoV-2 seronegative and seropositive TB patients showed a decline in IL-1ra over the six-month treatment period. This trend indicates immune restoration due to a reduced bacterial load and has been associated with a good treatment response (32, 33). However, at month six, IL-1β and IL-8 remained lower in SARS-CoV-2 seropositive TB patients, along with Basic FGF, which is known to increase in response to treatment (34). It is therefore clear that COVID-19 exerts long-lasting regulatory effects, which may delay resolution of immune perturbations in TB patients, though the effects appear to lessen over time. Future studies should monitor *Mtb* load to determine if these changes negatively impact clinical treatment response.

Although cytokines are reliable biomarkers of the immune response, our study was limited to unstimulated serum samples. A step forward would be to assess cytokine responses following TB/COVID-19 antigen stimulation to elucidate disease-specific changes and to identify any functional impairment. Moreover, future analyses should involve multivariable logistic regression to define cytokine signatures of difference. Correlation of cytokine profiles to clinical data, such as bacterial burden and chest x-ray score, will help to test hypotheses generated in this study, and to determine the clinical implications of our findings. Of note, it is well-established that the impact of COVID-19 on the immune system worsens with severity of acute infection (27), but this data was not available for our study. In addition, the described period of COVID-19 antibody waning is 6-8 months, so seronegative patients may have had COVID-19 outside of this timeframe (35). Our results warrant further studies to stratify SARS-CoV-2 seropositive patients based on date and severity of infection. This would shed more light on the determinants of the impact of prior COVID-19 on TB and ORD patients. It would also be interesting to assess the longevity of the functional impairment that we observed, to ascertain whether the effect is transient or long-term.

In conclusion, we have shown that past SARS-CoV-2 infection is associated with markedly reduced serum cytokine levels in both TB and ORD patients. SARS-CoV-2 seropositivity may therefore be used to anticipate reduced cytokine responses in these individuals. Our results provide evidence to suggest that COVID-19 may lead to an impaired response to TB and ORDs. Future work to determine if this increases risk of relapse or TB disease progression will be important in ascertaining the long-term impact of COVID-19 on *Mtb* immunity.

## Data availability statement

The raw data supporting the conclusions of this article will be made available by the authors, without undue reservation.

## Ethics statement

The studies involving humans were approved by MRC/Gambia government joint ethics committee. The studies were conducted in accordance with the local legislation and institutional requirements. The participants provided their written informed consent to participate in this study.

## Author contributions

AC: Formal analysis, Investigation, Methodology, Writing – original draft. IM: Investigation, Methodology, Supervision, Writing – review & editing. AG: Data curation, Investigation, Methodology, Writing – review & editing. AS: Investigation, Writing – review & editing. OC: Data curation, Software, Writing – review & editing. JM: Data curation, Investigation, Writing – review & editing. AB: Data curation, Methodology, Writing – review & editing. EC: Data curation, Methodology, Supervision, Writing – review & editing. GD: Methodology, Project administration, Resources, Writing – review & editing. SiB: Project administration, Resources, Writing – review & editing. SaB: Methodology, Resources, Writing – review & editing. OO: Methodology, Resources, Supervision, Writing – review & editing. JW: Funding acquisition, Writing – review & editing. GW: Conceptualization, Resources, Writing – review & editing. JS: Conceptualization, Funding acquisition, Resources, Writing – review & editing.
