# Supplementary material for: The impact of prior SARS-CoV-2 infection on host inflammatory cytokine profiles in patients with TB or other respiratory diseases
Source: Front Immunol. 2023 Dec 21;14:1292486. doi: 10.3389/fimmu.2023.1292486 (PMC10764540; doi:10.3389/fimmu.2023.1292486)
Supplement: Supplementary file 1 [file DataSheet_1.pdf]

Supplementary Material

Figures

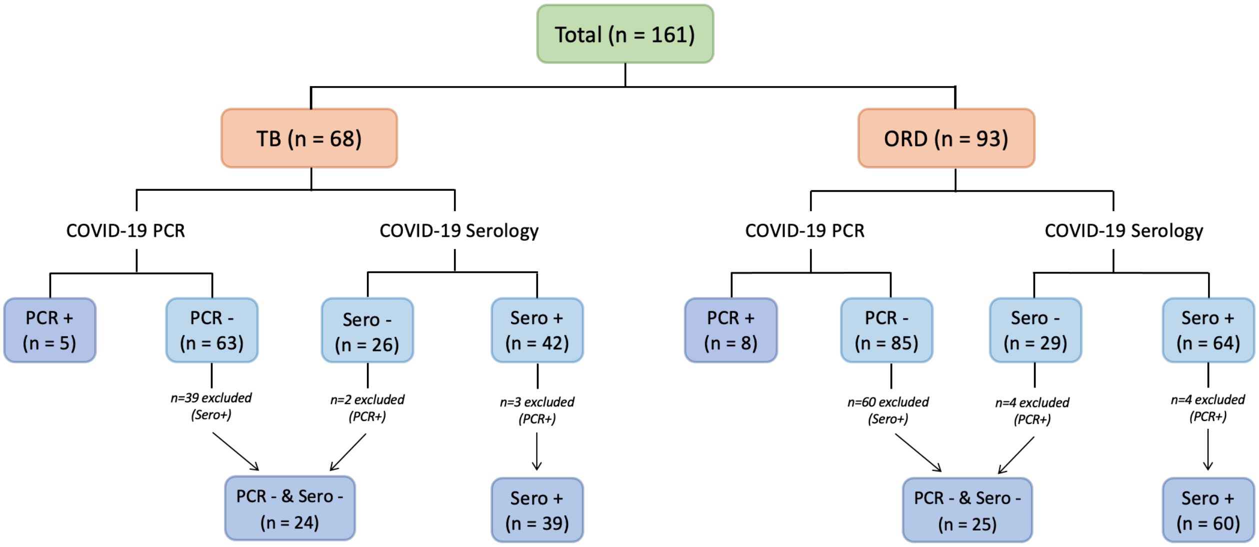

Supplementary Figure 1. Flowchart of the study population. All 161 participants were classified as having either TB or ORD, depending on their GeneXpert Ultra result or clinical diagnosis. Participants were then further divided according to their COVID-19 PCR and serology results.

Tables

Supplementary Table 1. Median serum cytokine concentrations in TB patients with (Sero+) and without (Sero-) prior COVID-19 infection

Cytokine	Median concentration [IQR] in Sero- (pg/mL)	Median concentration [IQR] in Sero+ (pg/mL)	P-value
Basic FGF	11.28 [9.9-13.2]	10.12 [8.5-12.3]	0.0597
Eotaxin	16.37 [6.8-35.7]	7.83 [2.4-18.1]	0.0547
G-CSF	84.64 [68.6-144.0]	62.48 [37.5-96.0]	**0.0126**
IL-1β	0.97 [0.24-1.28]	0.14 [0.14-0.62]	**0.0022**
IL-4	2.04 [1.59-2.61]	1.20 [0.94-1.69]	**0.0003**
IL-7	9.55 [2.9-16.4]	1.89 [1.3-7.2]	**0.0003**
IL-8	9.65 [1.6-23.1]	2.12 [1.0-7.0]	**0.0179**
IL-9	79.30 [16.6-171.7]	33.04 [20.6-70.1]	0.0524
IL-13	0.53 [0.37-1.29]	0.17 [0.17-0.50]	**0.0009**
IP-10	402.4 [104.7-1849]	119.8 [32.49-436.3]	**0.0126**
MCP-1	2.04 [0.29-4.09]	0.29 [0.29-2.38]	0.0487
MIP-1α	2.80 [1.39-5.53]	1.93 [0.96-2.60]	0.0385
MIP-1β	55.61 [17.0-80.2]	30.62 [23.5-47.2]	0.0529
PDGF-BB	1465 [267.1-3418]	483.6 [182.5-1051]	0.0447
RANTES	1989 [284.5-44758]	596.4 [105.3-1705]	0.0693
TNF-α	3.40 [1.68-9.38]	1.68 [1.68-3.34]	**0.0166**

Note: Sero+/- = seropositive/seronegative for SARS-CoV-2 antibodies; IQR = interquartile range. Significant differences ($p \leq 0.035$) are in bold. Sero+ n=39; Sero- n=24.
